# Reading the dyslexic brain: multiple dysfunctional routes revealed by a new meta-analysis of PET and fMRI activation studies

**DOI:** 10.3389/fnhum.2014.00830

**Published:** 2014-11-11

**Authors:** Eraldo Paulesu, Laura Danelli, Manuela Berlingeri

**Affiliations:** ^1^Department of Psychology, University of Milano-BicoccaMilan, Italy; ^2^NEUROMI- Milan Center for Neuroscience, University of Milano-BicoccaMilan, Italy; ^3^fMRI – Unit, Istituto di Ricovero e Cura a Carattere Scientifico GaleazziMilan, Italy

**Keywords:** developmental dyslexia, meta-analysis, fMRI, PET, ALE, hierarchical clustering

## Abstract

Developmental dyslexia has been the focus of much functional anatomical research. The main trust of this work is that typical developmental dyslexics have a dysfunction of the phonological and orthography to phonology conversion systems, in which the left occipito-temporal cortex has a crucial role. It remains to be seen whether there is a systematic co-occurrence of dysfunctional patterns of different functional systems perhaps converging on the same brain regions associated with the reading deficit. Such evidence would be relevant for theories like, for example, the magnocellular/attentional or the motor/cerebellar ones, which postulate a more basic and anatomically distributed disorder in dyslexia. We addressed this issue with a meta-analysis of all the imaging literature published until September 2013 using a combination of hierarchical clustering and activation likelihood estimation methods. The clustering analysis on 2360 peaks identified 193 clusters, 92 of which proved spatially significant. Following binomial tests on the clusters, we found left hemispheric network specific for normal controls (i.e., of reduced involvement in dyslexics) including the left inferior frontal, premotor, supramarginal cortices and the left infero-temporal and fusiform regions: these were preferentially associated with reading and the visual-to-phonology processes. There was also a more dorsal left fronto-parietal network: these clusters included peaks from tasks involving phonological manipulation, but also motoric or visuo-spatial perception/attention. No cluster was identified in area V5 for no task, nor cerebellar clusters showed a reduced association with dyslexics. We conclude that the examined literature demonstrates a specific lack of activation of the left occipito-temporal cortex in dyslexia particularly for reading and reading-like behaviors and for visuo-phonological tasks. Additional deficits of motor and attentional systems relevant for reading may be associated with altered functionality of dorsal left fronto-parietal cortex.

## Introduction

Developmental dyslexia (DD), the inability of acquiring fluent reading skills notwithstanding normal intelligence, adequate socio-cultural conditions, and preserved elementary sensory skills (DSM-IV, American Psychiatric Association, 1994; ICD-10, World Healt Organization, 1993), often co-occurs with phonological deficits (Snowling, 2001) that persist in adult life (Paulesu et al., 2001; Ramus et al., 2003). While it is fairly clear which classes of phonological tasks are more sensitive in bringing about a deficient performance in dyslexics (e.g., spoonerism tasks; see for example, Pennington et al., 1990), the fine-grained nature of cognitive deficits underlying these faulty performances remains to be established fully (Frith, 1999).

Subjects with DD may present a more complex behavioral profile (Menghini et al., 2010), the reading and phonological difficulties being sometimes accompanied by attentional, visual- and auditory-magnocellular and/or motor-cerebellar impairments (Facoetti et al., 2000; Nicolson et al., 2001; Stein, 2001; Gaab et al., 2007); these are hereafter called “*additional deficits*”^1^. The prevalence of the *additional deficits* may vary from sample to sample fuelling the debate on whether a core dyslexia syndrome exists together with a core underlying cognitive deficit. Indeed, the variable importance given to the *additional deficits* by different authors is one strong motivation for the presence of competing interpretations of dyslexia as a syndrome. The matter is complicated by the fact that the studies on co-morbidity in dyslexia have been run in groups selected with very different criteria: the range spans from studies on highly compensated adult university students in some cases (Ramus et al., 2003; Reid et al., 2007) to unselected young kids in other cases (Heim et al., 2008; Menghini et al., 2010). Studies in adult dyslexics have the advantage of permitting the assessment of a relatively stable neurocognitive system and to minimize the observation of co-occurring deficits due to delayed maturation; studies in kids are more prone to the uncertainties due to the—not necessarily synchronous—development of the multiple systems involved in reading and to the changing neuropsychological patterns that may place a given kid in the dyslexic or in the normal population range, depending on the year of testing (see for example, Shaywitz et al., 1992). Of course, studies in kids have the advantage of giving information relevant for the developmental process while the reading skill is being acquired.

There have been great hopes that functional anatomical studies of dyslexia could contribute to a better understanding of the disorder: it has been reasoned that if a well-defined malfunctioning brain system was identified, one could make stronger inferences on the nature of dyslexia at the cognitive level as well. This would have had obvious consequences in the field of rehabilitation (Demonet et al., 2004).

Indeed, brain imaging has had the merit of giving a demonstration that dyslexia has neurological bases. However, this demonstration has come sometimes in contrasting ways, giving further breath to the debate on the nature of dyslexia and on whether different forms of dyslexia exist and their relative weight.

By the time of the completion of the data collection for this paper, there have been more than 50 functional imaging papers on dyslexia that one could use for a formal review of the literature with a meta-analysis.

This previous literature can be grouped in few broad classes of activation studies: studies with tasks involving primarily reading (including lexical decision tasks, phonological awareness tasks or semantic tasks); lexical retrieval for visual stimuli, as in picture naming; studies on auditory phonological processes; studies on motor tasks and motor learning; studies on visual perception (picture or face oriented) or on visuo-spatial attention; studies on early visual or auditory processes, including stimuli tackling the magnocellular systems.

After such a huge experimental effort in the field, any review of the data based on a conventional verbose discussion of what is nominally described by the authors would prove insufficient, confusing, and sometimes contradictory. This is also because a nominal reference to a given brain structure, and the ensuing discussions, is deprived of much value and sometimes misleading when the precise stereotactic location of a statistical effect may point to more specific cortical or subcortical regions: congruence and incongruence of different data may only appear such because of this impreciseness^2^.

In addition, the relative weight of a given study, based on the sample sizes and statistical thresholds adopted, is often impossible to deal with. Having clearly in mind the aforementioned limitations of *verbose*, that is, non-quantitative, reviews, we mention hereafter those that seem to be the most solid findings for reading related tasks. To make this illustration, we use some of the raw data that were entered into a formal meta-analysis in the paper. Much of this discussion will hopefully be superseded by the results of the present meta-analysis whose aim was in fact to shed further light in the dyslexia imaging literature by showing findings that truly replicate across studies of the same class and perhaps across studies of different classes.

### Studies on the core symptoms: reading and phonological processing

The studies involving single-word reading in some form indicate dysfunction of both left occipito-temporal (ventral) (see Paulesu et al., 2001; Shaywitz et al., 2002 and more 20 studies from those listed in Table 1) or left temporo-parietal (dorsal) cortex (see for example Rumsey et al., 1992, 1997a). In particular, it has been proposed that the dorsal temporo-parietal cortex might be associated with an early dysfunction of phonological processing, emerging in the initial stage of learning process (Turkeltaub et al., 2003; Sandak et al., 2004), while the ventral occipito-temporal region may be associated with perturbed maturation of the word recognition systems (Paulesu et al., 2001; Sandak et al., 2004), a finding that generalizes across different alphabetic orthographies (Paulesu et al., 2001) and even Chinese (Hu et al., 2010).

**Table 1:** **List of papers included in the metanalysis**.

**Authors**	**Year**	**Tecnique**	**Sample size (Controls/Dyslexics)**	**Age of subjects**	**Modality presentation of stimuli**	**Experimental task**
Bach et al.	2010	fMRI	18/11	Children	Visual	Letter substitution
Backes et al.	2002	fMRI	8/8	Children	Visual	Line orientation, string comparison, non-word reading, semantic judgment
Beneventi et al.	2009	fMRI	13/11	Children	Visual	Sequential verbal working memory task
Beneventi et al.	2010b	fMRI	14/12	Children	Visual	Working memory n-back task
Beneventi et al.	2010a	fMRI	13/11	Children	Visual	Working memory n-back task
Booth et al.	2007	fMRI	39/39	Children	Auditory, visual	Word-pair semantic judgment
Brambati et al.	2006	fMRI	11/13	Adults	Visual	Word and pseudoword reading
Brunswick et al.	1999	PET	6/6	Adults	Visual	Word and pseudoword reading
Cao et al.	2006	fMRI	12/12	Children	Visual	Word rhyming
Cao et al.	2008	fMRI	14/14	Children	Visual	Word rhyming
Conway et al.	2008	fMRI	11/11	Adults	Auditory	Auditory pseudoword segmentation
Desroches et al.	2010	fMRI	12/12	Children	Auditory	Auditory rhyming task
Dufor et al.	2007	PET	16/14	Adults	Auditory	Phoneme categorization
Eden et al.	2004	fMRI	19/19	Adults	Auditory	Word repetition, phoneme delection
Gaab et al.	2007	fMRI	23/22	Children	Auditory	Sound discrimination
Georgiewa et al.	1999	fMRI	17/17	Children	Visual	Non-word reading, frequent word reading, phonological manipulation
Grande et al.	2011	fMRI	25/20	Children	Visual	Picture naming, reading aloud of words
Grunling et al.	2004	fMRI	21/17	Children	Visual	Slash pattern matching, letter strings matching, pseudoword matching, frequent word matching, pseudoword rhyming
Heim et al.	2010	fMRI	20/16	Children	Auditory, visual	Phoneme discrimination, motion detection, attention shifting, auditory discrimination of verbal and non-verbal stimuli
Hoeft et al.	2006	fMRI	20/10	Children	Visual	Word rhyming
Ingvar et al.	2002	PET	9/9	Adults	Visual	Word and pseudoword reading
Kast et al.	2011	fMRI	13/12	Adults	Auditory, visual	Lexical decision
Kovelman et al.	2011	fMRI	12/12	Children	Visual	Word matching, word rhyming
Kronbichler et al.	2006	fMRI	15/13	Children	Visual	Sentence reading
Kronschnabel et al.	2013	fMRI	22/13	Children	Visual	Word and pseudoword reading
Landi et al.	2010	fMRI	13/13	Children	Visual	Word rhyming, semantic categorization
MacSweeney et al.	2009	fMRI	7/7	Adults	Visual	Picture matching, word Rhyming
Maurer et al.	2011	fMRI	16/11	Children	Visual	Word matching, pseudoword matching, picture matching
McCrory et al.	2000	PET	8/6	Adults	Auditory	Word and pseudoword repetition
McCrory et al.	2005	PET	10/8	Adults	Visual	Word reading, pitcure naming
Menghini et al.	2006	fMRI	14/14	Adults	Visual	Implicit motor learning
Meyler et al.	2008	fMRI	12/23	Children	Visual	Sentence judgment
Monzalvo et al.	2012	fMRI	23/23	Children	Visual	Houses, faces, word and checkboard perception, sentence listening in native and foreign language
Nicolson et al.	1999	PET	6/6	Adults	Auditory	Sequence motor learning
Olulade et al.	2012	fMRI	12/9	Adults	Visual	Word and pseudoword rhyming, Spatial rotation
Paulesu et al.	1996	PET	5/5	Adults	Visual	Syllable rhyming, verbal short-term memory task
Paulesu et al.	2001	PET	36/36	Adults	Visual	Word and non-word reading
Pecini et al.	2011	fMRI	13/13	Adults	Audiovisual	Rhyme-generation task
Pekkola et al.	2006	fMRI	10/10	Adults	Audiovisual	Perception of matching and conflicting audio-visual speech
Peyrin et al.	2011	fMRI	12/12	Children	Visual	Letter matching
Reilhac et al.	2013	fMRI	12/12	Adults	Visual	Letter string comparison
Richlan et al.	2010	fMRI	18/15	Adults	Visual	Phonological lexical decision
Rimrodt et al.	2009	fMRI	15/14	Children	Visual	Word recognition, sentence judgment
Ruff et al.	2002	fMRI	11/6	Adults	Auditory	Implicit categorial perception of phonemes
Rumsey et al.	1997b	PET	14/17	Adults	Visual	Irregular word and pseudoword reading, phonologial and ortographic lexical decision
Schulz et al.	2008	fMRI	22/12	Children	Visual	Sentence judgment
Schulz et al.	2009	fMRI	30/15	Children	Visual	Sentence judgment
Steinbrink et al.	2012	fMRI	16/17	Adults	Auditory	Same-different judgment of vowel duration
Temple et al.	2000	fMRI	8/10	Adults	Auditory	Sound discrimination
Temple et al.	2001	fMRI	15/24	Children	Visual	Letter matching, letter rhyming
van der Mark et al.	2009	fMRI	24/18	Children	Visual	Phonological lexical decision
Vasic et al.	2008	fMRI	13/12	Adults	Visual	Verbal working memory task
Wimmer et al.	2010	fMRI	19/20	Adults	Visual	Phonological lexical decision

**Figure 1:**
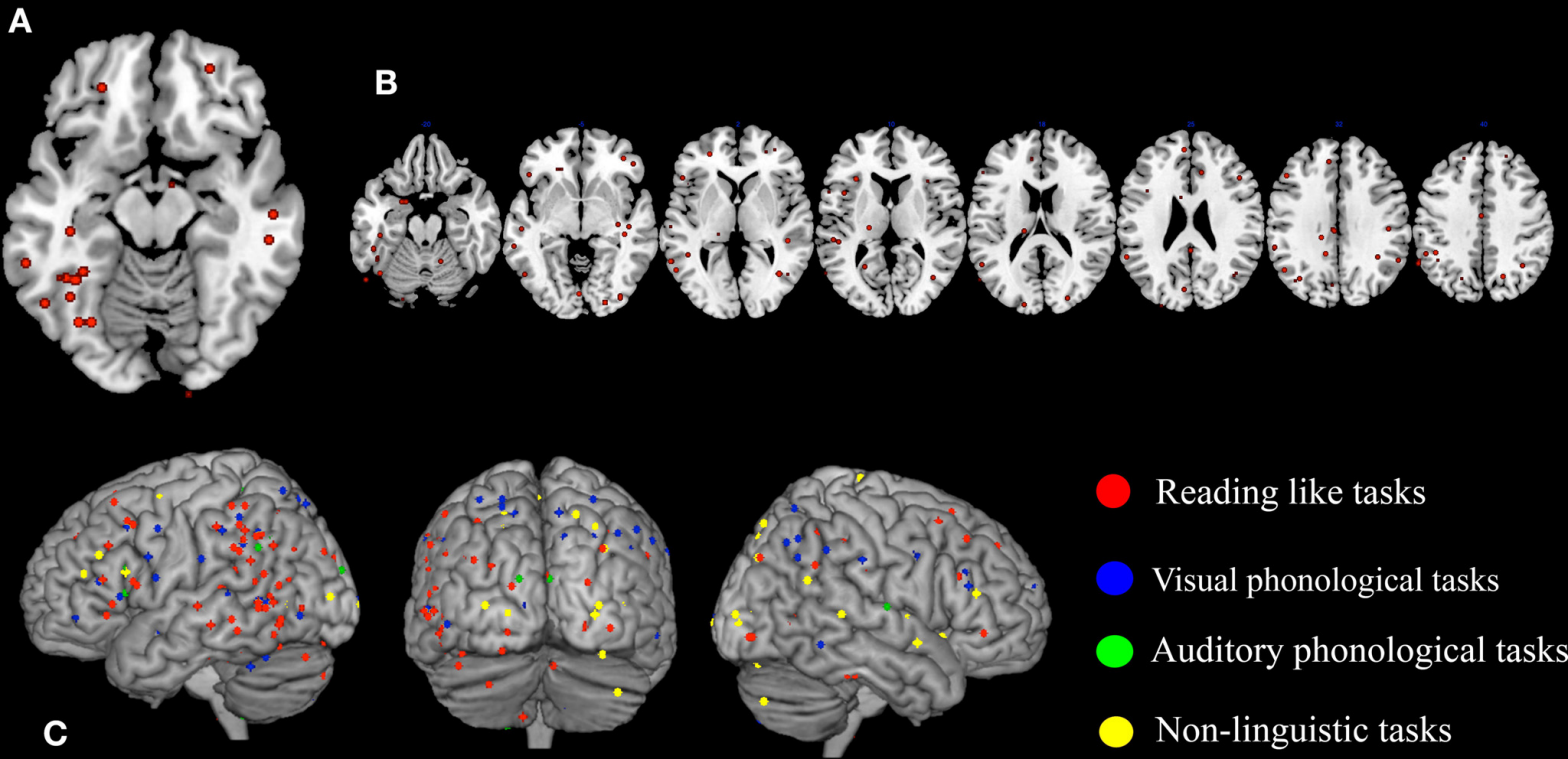
**Peaks of reduced activations in dyslexia for all tasks that involved reading (circles in red), for visual or auditory phonological tasks (circles in blue and in green, respectively) and for non-linguistic tasks (circles in yellow)**. **(A)** Show the highly replicated reduction of dyslexics at the level of the left ventral occipito-temporal peaks reported in literature. In **(B)** all the peaks of reduced activations observed in dyslexics during reading tasks included in our meta-analysis are reported. Finally, in **(C)** all the peaks of reduced activations observed in dyslexics during reading, phonological and non-linguistic tasks included in our meta-analysis are reported.

A first look on the highly replicated finding for the left ventral occipito-temporal cortex can be seen in Figure 1A, where the location of the local maxima of significant hemodynamic response reduction was described for dyslexics. In the figure we visualize the peaks of reduced activations in dyslexia for all tasks that involved reading from the papers listed in Table 1.

A further look to the distribution of the areas of reduced activations for any task involving reading in dyslexia (see Figure 1B), however, provides a more complex picture that clearly justifies the urge for a formal re-assessment of the data.

Are these patterns age dependent? Are some of them task dependent? What is the role of the right hemispheric hypo-activations for a behavior like reading that is highly dependent on a left-lateralized neural system (Cattinelli et al., 2013a; Taylor et al., 2013)? More importantly, what is the level of replication of the findings of any given paper? Is this seemingly highly distributed pattern of malfunction undermining our understanding of the biology and the cognition of dyslexia? These are all questions that are still in search of some formal answer.

In fact, the situation appears immediately more complex if one also considers phonological tasks, both visual and auditory. As one major theory of dyslexia predicates a phonological deficit it becomes logical to expect a great anatomical congruence between findings based on reading and findings based on phonological tasks. The way these focal effects (regional hypoactivations in dyslexia) overlap with the reading ones is illustrated by Figure 1C (dots in blue and dots in green). Clearly there is some degree of overlap between the three sets of findings.

However, there are also quite a few discrepancies. The same unsolved questions listed before apply here.

### *Ashes to ashes, noise to noise*: the contribution of the imaging findings on the additional deficits in dyslexia

Studies on what we call the additional deficits of dyslexia investigated the neural dysfunction of more basic abilities such as those of the magnocellular (visual or auditory) system, of the spatial attention system and of motor control with particular attention to the functions of the cerebellum.

The case of the visual magnocellular and visuo-motion perception system is an exemplar one: a dysfunction of this system is suggested by evidence that dyslexic may have reduced contrast sensitivity at the low spatial frequencies and low luminance levels (stimuli favored by the magnocells; Stein and Walsh, 1997), reduced visual-motion sensitivity, in particular for coherent motion (Cornelissen et al., 1995), that correlates with impaired letter position encoding (Cornelissen et al., 1998); the same deficit may explain greater crowding effects in dyslexic subjects (Zorzi et al., 2012). In addition, subjects with dyslexia may have subtle signs of a dysfunctional visuo-spatial attentional system (Facoetti et al., 2000) that may be more severe for the left hemispace in a sort of mini-neglect (Hari and Renvall, 2001; Liddle et al., 2009). This evidence was supported initially by ERP data (Galaburda and Livingstone, 1993) who found reduced VEPs in 5 dyslexics for low contrast reversing checkerboard stimuli (a finding not replicated by others—see Johannes et al., 1996) and a disorganized magnocellular subdivision of the lateral geniculate nucleus.

Initial fMRI evidence pointing to a dysfunctional magnocellular system was provided by Eden et al. (1996) followed by Demb et al. (1998) in two small samples of subjects: they found reduced activation of the visual motion area MT/V5, a result that was lately not confirmed by MEG data, as Vanni et al. (1997) found normal MT/V5 activation for moving stimuli.

Notwithstanding that the contribution of the magnocellular and visuo-motion perception system in normal reading remains contentious (no involvement of MT/V5 is seen for single word reading in normal subjects), the aforementioned results have been seen as a imaging evidence of the malfunction of the visual magnocellular system in dyslexia. Indeed, the magnocellular hypothesis remains a much pursued research avenue in dyslexia. Similar considerations may apply to the cerebellar hypothesis and its investigation.

To make this brutal introductory overview even more dismaying, Figure 1C (dots in yellow) shows how focal hypo-activations spread all over the brain if one considers non-linguistic tasks for either the visual modality or the motor one. This picture is quite similar with what would emerge if the scars and dyslaminations originally described by Galaburda et al. (1985) were superimposed onto the lateral surface of the brain in stereotactic space.

It should be noted that in these examples, we illustrate only voxels showing significant differences between groups. There is much more to be displayed if one considers as we did in the paper, also within group effects.

Clearly, such body of data cannot be assessed and summarized by a mere discussion of what has found paper X as opposed to paper Y. The obvious alternative to qualitative reviews is provided by formal meta-analyses, as their quantitative approach makes them more rigorous and less prone to subjective bias. In brain imaging, meta-analyses are generally used to identify groups of regional effects that fall sufficiently close in stereotactic space to be interpreted as reflecting a common functional-anatomical entity (Fox et al., 1998; Wager et al., 2007; Cattinelli et al., 2013b). The functional significance of any of these entities then needs to be analyzed, on the basis of the background information about the experiments that generated the activation peaks constituting them. Several meta-analytic studies, differing in the specific technique employed and the investigated cognitive domain, have appeared in the literature in recent years (Salimi-Khorshidi et al., 2009; Kober and Wager, 2010; Radua and Mataix-Cols, 2012). Quantitative meta-analytic approaches were also recently used to determine consistency across neuroimaging studies and to identify regions reported as dysfunctional in developmental dyslexia (Maisog et al., 2008; Richlan et al., 2009). In particular, two studies, using the Activation Likelihood Estimation (ALE) method (Maisog et al., 2008; Richlan et al., 2009), analyzed the neural differences between controls and dyslexics during reading and reading-related tasks, i.e., letter matching, rhyming, semantic judgment, and lexical decision tasks. In both articles, the authors suggested that developmental dyslexia is associated with the hypoactivation of the left occipito-temporal, temporo-parietal, and inferior frontal regions. No evidence for a systematic hyperactivation in the dyslexics was found (for the left inferior frontal cortex, nor for the cerebellum, as initially suggested by Shaywitz et al. (1998).

To provide information on the developmental progression of neural dysfunction in dyslexia, Richlan et al. (2011) performed a second meta-analysis and separated adult-related activations and children-related activations while comparing controls and dyslexics. They observed that the left occipito-temporal and temporo-parietal hypoactivation was present in the studies on adults. A hypoactivation was also observed in the anterior portion of the left occipito-temporal cortex for dyslexic children.

## Aims of the study

These previous meta-analyses were focused on the task of reading or on reading-like behaviors. Aim of this study was to further assess the dysfunctional anatomical correlates of dyslexia, to approach the issue of co-occurrence of neural dysfunction dyslexia and test the hypothesis that, beyond well replicated findings (the lack of commitment to reading of the left ventral occipito-temporal cortex), other functional anatomical deficits might be present. The usual logic used to test this hypothesis in previous studies has been to assess the presence of focal hypoactivations in non-reading tasks, for example, in motor learning (Nicolson et al., 1999; Menghini et al., 2006) or visual motion perception (Eden et al., 1996). Conversely, the logic behind our study is similar to the one of Danelli et al. (2013) for normal reading: given the vast literature supporting the involvement of multiple systems in dyslexia (Frith, 1999; Nicolson et al., 2001; Snowling, 2001; Stein, 2001; Reid et al., 2007; Pernet et al., 2009), and given that these systems normally intersect in the brain into higher order cortices (Danelli et al., 2013), we expected that, on top of differences in brain areas that are highly specific for reading, dyslexics may also show a more limited functional anatomical intersection between different systems normally overlapping in skilled readers. This would be revealed in the present meta-analysis by reduced presence of regional effects from dyslexic groups in clusters showing a mix of peaks from reading-like and non-reading-like behaviors in normal controls.

In the present study this hypothesis was tested using a meta-analytical approach based on the optimized hierarchical clustering (HC) algorithm of Cattinelli et al. (2013b), complemented by the ALE algorithm (Turkeltaub et al., 2002; Eickhoff et al., 2009).

Hierarchical clustering has the advantage of permitting *post-hoc* statistical assessments of the functional or group assignations of individual clusters without the constraint of considering super-homogenous tasks at the stage of cluster identification, as when using ginger-ALE alone.

**Figure 2:**
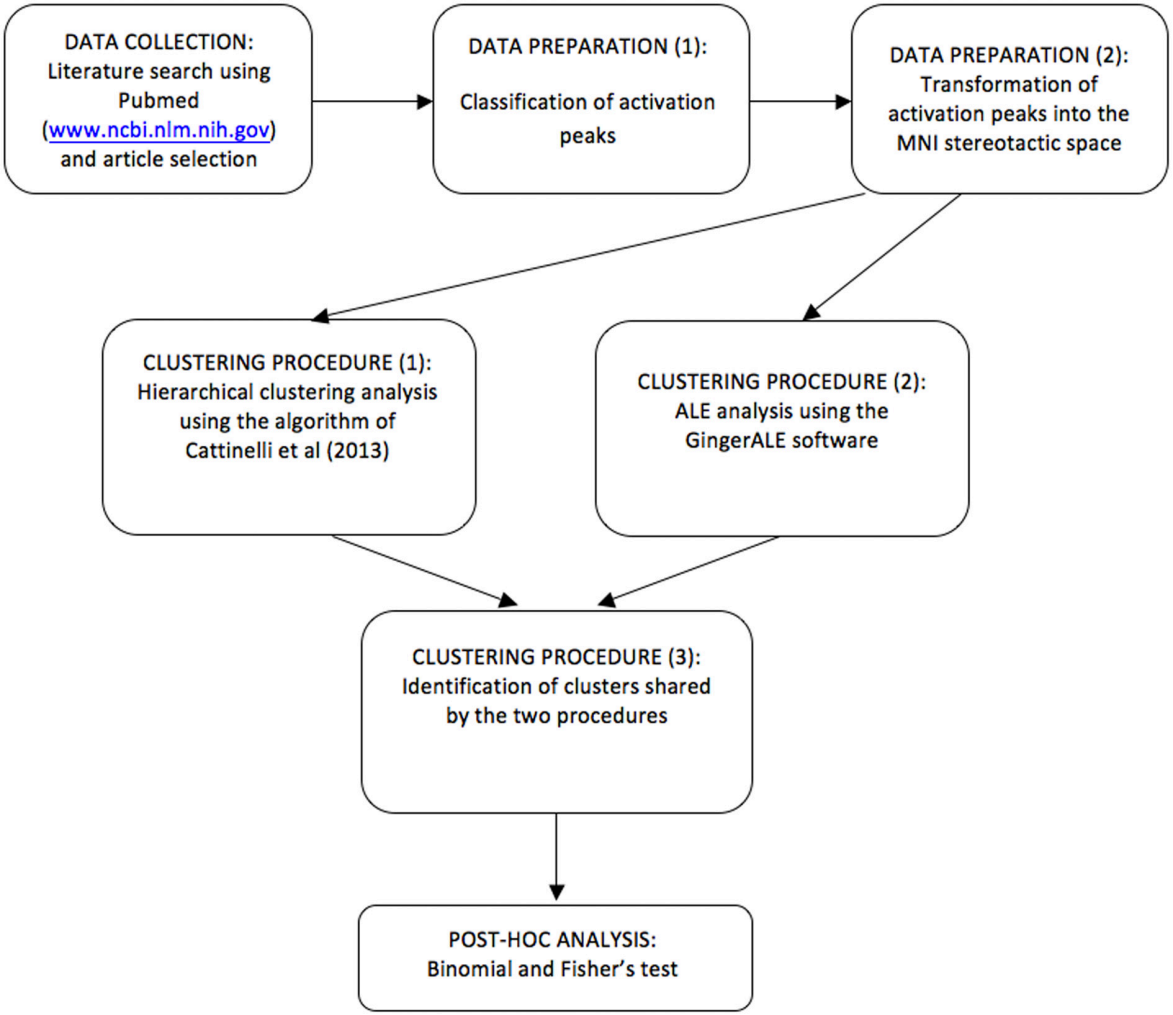
**A schematic flowchart diagram showing the procedure by which data are selected, clusters are estimated, tested and classified using HC and ALE**.

However, hierarchical clustering does not provide a statistical test of the spatial significance of a given cluster against a random reference distribution of regional effects. This is permitted by the ALE approach (Turkeltaub et al., 2002; Eickhoff et al., 2009) that we used to complement our analyses. A schematic flowchart diagram is now reported in Figure 2. A previous example of this combined approach can be found in Crepaldi et al. (2013), where, in addition to the dual meta-analytical procedure, the clusters were assessed *post-hoc* not only for simple effects but also for interaction effects, as in the present study.

By considering all imaging studies on dyslexia, no matter the neurocognitive domain under investigation, we hoped to detect the existence of a systematic co-occurrence of dysfunctional patterns of different functional systems and to evaluate whether these involve different system specific brain regions or rather multimodal regions that normally show intersections of multiple systems. The face validity of the latter hypothesis was also assessed by comparison with the data of Danelli et al. (2013).

## Methods

### Data collection and preparation

Our meta-analysis is based on 53 neuroimaging articles investigating the anatomofunctional dysfunction of developmental dyslexia using PET or fMRI in both children and adult subjects published to September 2013.

Studies were selected through PubMed database (http://www.ncbi.nlm.nih.gov/pubmed/) running five queries. The search keys were: “Dyslexia AND fMRI,” “Dyslexia AND PET,” “Dyslexia AND neuroimaging,” “Dyslexia AND functional Magnetic Resonance Imaging,” and “Dyslexia AND Positron Emission Tomography.” These queries returned 544, 34, 462, 267, and 45 entries, respectively.

After removing duplicates, we included only studies that did satisfy the following inclusion criteria: (1) sample population composed of both normal controls and subjects with developmental dyslexia; (2) imaging technique: PET or fMRI; (3) whole brain voxel based data-analyses using stereotactic conventions; region-of-interest analyses were not considered nor multiple single case analyses restricted to few regions, as, for example, in Eden et al. (1996); (4) presence of data for either within group comparisons, or between group comparisons or both.

For the suitable studies, in the meta-analysis we used data derived from (i) within group *simple effects* and (ii) between *group comparisons*. We incorporated also the within group data to have a more complete survey on whether a given brain region was differentially activated across groups, while still being active in each a group above a given conventional threshold, or whether the region, besides being significantly associated with one group, it never reached statistically significant effects in the other group. In any event, for the interaction group-by-task effects we only considered 1st order interactions.

Only data emerging from univariate statistical analyses were considered.

By applying such criteria, we included 2360 stereotactic activation loci, 1402 associated with controls and 958 associated with subjects with dyslexia. Thirty-nine foci were excluded by the analyses because they were outside of the boundaries the MNI stereotactic space.

The main characteristics of the 53 experiments included in this meta-analysis are reported in Table 1.

### Classification of the raw data prior to clustering analyses

For each activation peak, we recorded all relevant information about the statistical comparison that generated it. We therefore determined a list of classification criteria to characterize each peak of activation included in the dataset (Table S1 of the Supplementary Materials). These classifications were used for initial *post-hoc* statistical comparisons on the clusters that passed the ginger-ALE test for spatial significance.

- *Subjects*: we classified each peak on the basis of the age of the subjects. In particular, we considered as separate categories in this variable: (i) *children* when sample age was under the age of 18, (ii) *adults* when sample age was above the age of 18.
- *Stimulation modality*: (i) *auditory* or (ii) *visual*.
- *Nature of the experimental task*: for the sake of simplicity, the data were grouped in the following two broad categories: (i) *reading-like tasks* included orthographic tasks as letter matching or string comparison, reading, visual lexical decision, visual phonological tasks as rhyming, word manipulation or verbal short-term memory tasks, visual semantic tasks as written sentence comprehension or semantic judgment; because of its functional analogy (see McCrory et al., 2005) with the reading behavior and the documented reduced activations in reading-related brain areas in developmental dyslexia, picture naming was included in this group (ii) *non-reading-like tasks* included auditory perception task as sound, vowel or speech discrimination, motor tasks, visual perception and visuo-spatial attention tasks and lip reading for single vowel sounds^3^.

For each peak we also completed our database with information about the variables listed below.

- *Scanning Technique* (PET or fMRI),
- *Stereotactic template* (*MNI* or *Tailarach and Tournoux* template),
- *Staistical thresholds* and *nature of the correction* for multiple comparisons.

To make it possible a combination of data coming from studies based on different stereotactic spaces, the stereotactic coordinates of studies in which activation peaks were reported in terms of the Talairach and Tournoux atlas (Talairach and Tournoux, 1988) were transformed into the MNI (Montreal Neurological Institute) stereotactic space (Mazziotta et al., 1995); the transformation was done using the software GingerALE, using MatthewBrett's script (http://imaging.mrc-cbu.cam.ac.uk/imaging/MniTalairach).

### Clustering procedure

First, we performed a hierarchical clustering analysis (HC) of the activation peaks as described in Cattinelli et al. (2013b): the analysis allowed us to extract the principal clusters of regional effects from the database. Hierarchical clustering was performed by using functions implemented in MATLAB 7. After computation of squared euclidean distances between each pair of the input data, clusters with minimal dissimilarity were recursively merged using Ward's (1963) criterion which minimizes total intracluster variance after each merging step. As described in Cattinelli et al. (2013b) and Crepaldi et al. (2013), “this procedure results in a tree, whose leaves represent singletons (i.e., clusters formed of a single activation peak), and whose root represents one large cluster including all the activation peaks submitted to the algorithm. Each level of the tree reports the clusters created by the algorithm at a specific processing step, as it progresses from individual activation peaks at the lowest level to the all-inclusive final cluster at the top of the tree”. The procedure was continued until the average standard deviation around the cluster centroids of the individual peaks, in the *x, y*, and *z* directions, remained below 7.5 mm. This measure roughly mimics the spatial resolution of fMRI studies. As hierarchical clustering may be sensitive to the order in which the individual data are processed, and may generate alternative clustering trees (Morgan and Ray, 1995), an optimal clustering solution was identified by accepting the solution with maximized the between cluster error sum of squares (see Cattinelli et al., 2013b).

The mean coordinates of each cluster included in the final set were then passed as an input to a MATLAB script to automatically label the anatomical correspondence of the stereotactic coordinates of the centroids of each cluster. This procedure implied a query of the Automatic Anatomical Labeling (AAL) template available in the MRIcron visualization Software (Rorden and Brett, 2000).

HC analyses have the advantage of permitting *post-hoc* assessment of the functional meaning of a given cluster (see for example, Cattinelli et al., 2013a; Crepaldi et al., 2013) or, as in the present study, its assignation to a class of subjects (e.g., clusters specific or preferentially associated with controls rather than clusters associated with dyslexics).

However, HC does not quantify the significance of each individual cluster with reference to the probability of a spatially distributed statistical process. To protect ourselves from considering clusters of limited biological significance, the spatial distribution of the clusters identified by HC was compared with the results of a different meta-analytical method, namely the Activation Likelihood Estimation technique as implemented in the GingerALE software (Eickhoff et al., 2009; Turkeltaub et al., 2012). Only clusters also present in the GingerALE analyses were further considered (the threshold was set at *p* < 0.05 with FDRpN correction).

### *post-hoc* statistical analyses on the resulting clusters

Group, age, or task preferential associations were assessed with the binomial test as follows.

For the group effect, we tested whether the distribution of control- and dyslexic-related peaks within each cluster was significantly different from the overall proportion of control- and dyslexic-related peaks included in the whole sample of coordinates (1382/2321 = 0.59543 for controls and 939/2321 = 0.40457 for dyslexics). To this end, we used the binomial distribution and computed the probability of observing a specific number of peaks associated with a given group as the number of successes in a series of independent randomly-distributed trials: when this probability was below 0.05, the cluster was considered to be associated with either the control or dyslexic groups.

Similar analyses were implemented in these clusters to test their association with either reading-related or non-reading related tasks and with either children or adult group. The proportion of non-reading and reading-related peaks included in the whole sample of coordinates was 406/2321 (=0.17492) and 1915/2321 (=0.82508), respectively, while the proportion of children- and adult-related peaks included in the whole sample of coordinates was 1144/2321 (=0.49289) and 1177/2321 (=0.50711), respectively^4^.

We also assessed whether there were interactions effects within each cluster: the group-by-task and group-by-age interactions were tested with Fisher's exact test (Fisher, 1970); this estimates whether the distribution of one categorical variable (group) varies according to the levels of a second categorical variable (experimental task or age class), thus revealing clusters that were associated with either group in one task (e.g., reading-like tasks in controls), but with the opposite group in another task (e.g., dyslexic in non-reading-like tasks).

The odds ratio under the null hypothesis of the Fischer's test on the individual clusters was corrected to reflect the distribution of the categories under examination in the entire data-set. The odd ratio for group-by-task interaction was 1.09, for group-by-age interaction was 0.81 and for task-by-age interaction was 1.27.

Some of the interaction effects were tested to replicate previous analyses published in other meta-analytical papers: for example, the age-by-group interaction described by Richlan et al. (2013). We believe that describing these results, even if not all discussed in detail later on, leaves an important trace behind this paper for future assessments.

Finally, clusters that did not show a significant group preferential association were assigned to a class called undifferentiated. Among these clusters, we attempted to highlight those having higher probability of actually being completely non-specific, by performing binomial tests along the group axis. In particular, we assumed that clusters whose one-tailed *p*-value was greater than 0.5 for both groups are of high chance of being genuinely non-specific.

All post-clustering statistical analyses were performed using the free statistical software *R* (the code is available upon request to Manuela Berlingeri).

### Comparison with Danelli et al. (2013) mapping of reading and systems involved in dyslexia

The results of the clustering analyses were also compared with the independent fMRI data described by Danelli et al. (2013). In that paper, the authors described fMRI patterns of intersection between the normal reading system and the auditory phonological system, or the visual motion/magnocellular system, or the motor/cerebellar system: they also reported reading per-se activations, that is, areas activated for single pseudowords reading, once any trend for the other aforementioned tasks was excluded by the analysis^5^. Comparison with this independent data-set helped in the interpretation of the functional relevance of the data of the present meta-analysis.

### Additional analyses

The paucity of data on MT/V5 due to the lack of group-based data would inevitably dismiss the MT/V5 finding: to avoid this, we identified the average MT/V5 of the normal controls in Eden et al. (1996) and looked for the closest cluster in the meta-analysis.

Finally, the data of the regional effects associated with non-reading-like tasks (e.g., motor tasks, attentional tasks, visual perception tasks etc.) were also submitted to a separate meta-analysis. This additional meta-analysis was motivated by the desire of excluding the possibility that the overwhelmingly larger number of peaks from the reading-like experiments (# of peaks: 1915) could have masked the manifestation of specific clusters from the *non-reading-like* data (# of peaks: 406)^6^. As above, we tested whether the distribution of control- and dyslexic-related peaks within each cluster was significantly different from the overall proportion of control- and dyslexic-related peaks included in the whole sample of coordinates (0.58 for controls and 0.42 for dyslexics). To this end, we used the binomial distribution and computed the probability of observing a specific number of peaks associated with a given group as the number of successes in a series of independent randomly-distributed trials: when this probability was below 0.05, the cluster was considered to be associated with either the control or dyslexic groups. These analyses were performed only on clusters that showed a spatial congruence in the HC and ALE procedures.

## Results

**Figure 3:**
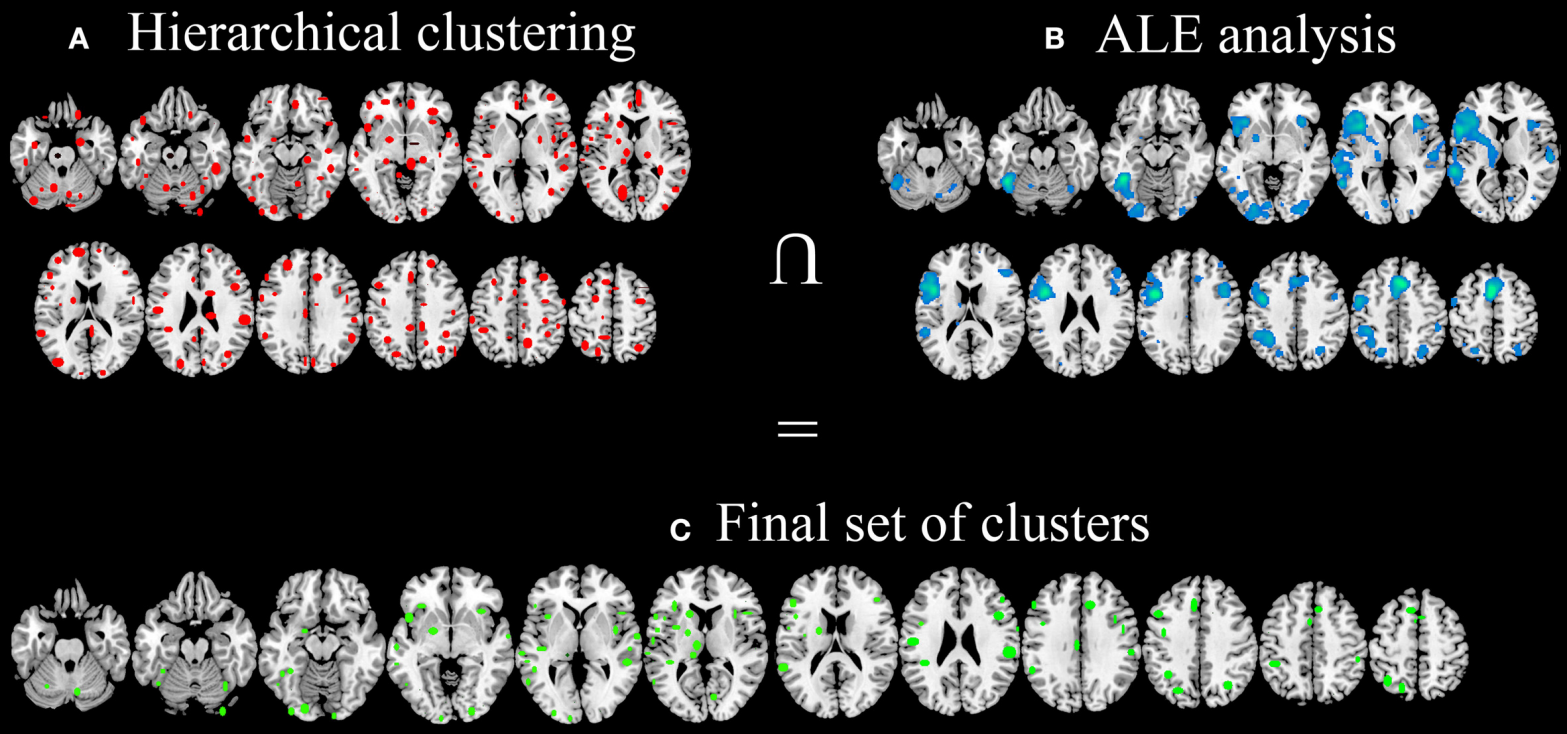
**Clusters identified with HC (A), clusters identified using ALE approach (B) and the final data-set of clusters, identified in both HC and ALE meta-analyses and considered for *post-hoc* statistical analyses (C)**.

The hierarchical algorithm identified a total of 193 clusters (Figure 3A)—96 clusters in the left hemisphere and 97 ones in the right hemisphere—with 2 to 51 peaks each, from 2 to 18 different studies; mean standard deviation along the three axes were 4.54 mm (x-axis), 4.83 mm (y-axis), and 4.76 mm (z-axis).

After the comparison of these results with ALE maps (Figure 3B), only 92 out of 193 clusters (Figure 3C) were considered for subsequent analyses.

### Group-preferential clusters

When we indicate a cluster as “related to” or “preferential for” a group, we imply that there was a significantly greater proportion of peaks in one group as opposed to the other. This would correspond to the terminology suggested by Pernet et al. (2007), the so-called “preferential response” for brain regions with a comparatively greater response in a given condition/group, with no zero response in the control condition/control group. Nine clusters were preferentially associated with controls, while five clusters were associated with dyslexics (Table 2). The peaks distribution for each significant cluster is reported in the contingency tables in Supplementary Materials (Table S2).

**Table 2:**
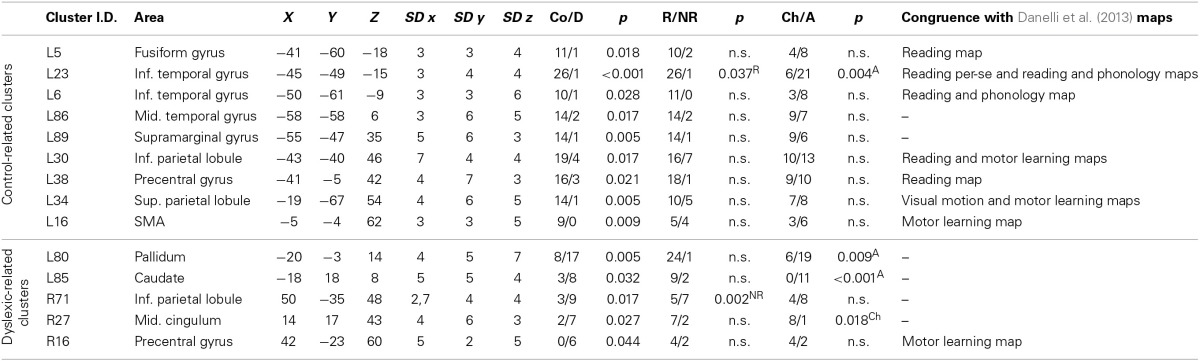
**Clusters with significant group specificity**.

*Co, controls; D, Dyslexics; NR, non-reading-like tasks; R, reading-like tasks; A, Adults; Ch, Children*.

#### Clusters associated with normal readers

**Figure 4:**
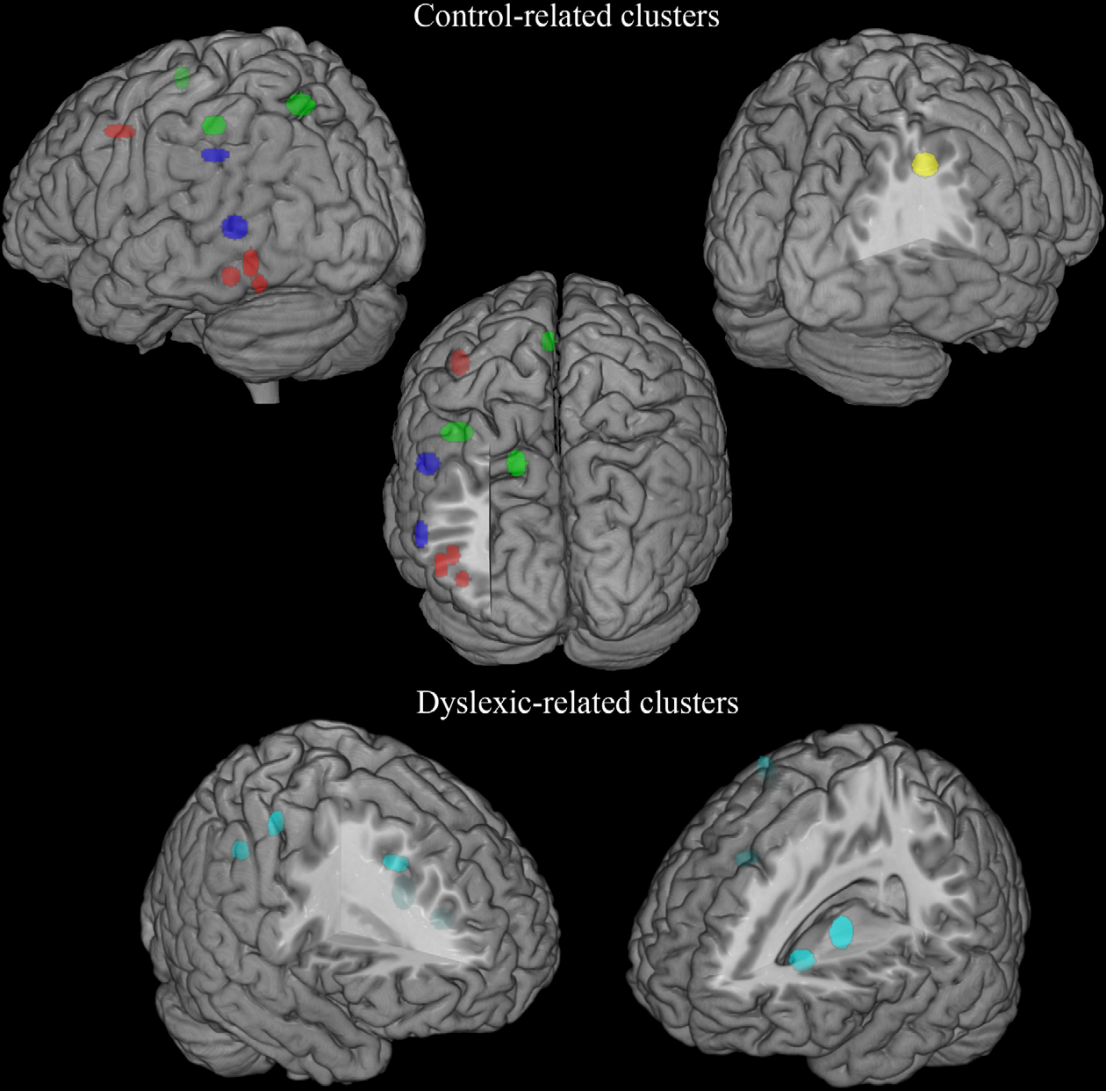
**Distribution of group-related clusters that showed a spatial congruence in the HC and ALE procedures**. The red dots represent the control-related clusters that fell in reading and phonological specific activations in Danelli et al. (2013), the blue dots represent the control-related clusters that were not observed in Danelli et al. (2013) and the green dots represent the control-related clusters fell in visual motion and motoric activations in Danelli et al. (2013). The right yellow dots represent the control-related cluster identified by the meta-analysis restricted to the non-reading-like tasks. Finally, dyslexic-related clusters are reported in cyan.

There was a distributed left ventral occipito-temporal network involving the infero-temporal (clusters L6, L23) and fusiform (cluster L5) regions (areas in red in Figure 4). Within this network, peaks coming from lexical decision tasks were present in cluster L23 only, while all other reading-like behaviors were fairly evenly present in the entire set of the three left occipito-temporal clusters. Once compared with Danelli et al. (2013) statistical maps, the cluster L5 fell in the reading network while L6 fell in a region of the shared activations by an auditory phonological task and reading. L23 fell in between these two regions.

Moving toward more dorsal regions, there were two areas in the middle temporal and supra-marginal gyri (L86 and L89; areas in blue in Figure 4) that were associated with the normal controls for a mixture of tasks including reading but also active phonological manipulation tasks, involving some working memory demands.

Even more dorsally, we also found a left hemispheric network involving the posterior part of the supplementary motor cortex, the superior parietal cortex, the dorsal portion of the inferior parietal lobule (areas in green in Figure 4): these clusters included peaks from tasks involving phonological manipulation (e.g., phonological short-term memory), but also motoric or visuo-spatial perception/attention. A comparison with Danelli et al. (2013) data confirmed the mixed nature of the functional properties of these regions, which were involved in motoric tasks and, for the superior parietal region, in the visual motion perception task as well.

#### Clusters associated with dyslexic readers

These included the left basal ganglia (head of the caudate; pallidum), the right anterior cingulate, right precentral cortex and the right inferior parietal lobule (areas in cyan in Figure 4). While the left subcortical regions were brought about by reading-like tasks, the right hemispheric ones were associated with a variety of tasks, often of the non-reading-like kind.

Detailed description of group-related clusters is reported in Table 2.

### Task-preferential clusters

Four clusters, located in the opercular parts of the left inferior frontal gyrus, in the left insula and in the posterior portion of the left inferior temporal gyrus, were preferential for reading-like group, while five clusters, located in the right superior and inferior parietal lobule, in the superior temporal cortex, bilaterally, and in the left middle temporal gyrus, were significantly related with non-reading-like tasks.

### Age-preferential clusters

Fifteen clusters were preferentially associated with adults, while 10 clusters were associated with children. In particular, adult-related clusters were located in the left SMA, in the opercular part of the inferior frontal gyrus, bilaterally, in the left insula, in the left superior and inferior temporal gyrus, in the cerebellum, bilaterally, in the left pallidum and caudate nuclei, and in the left thalamus.

Children-related clusters were located in the pre-SMA, bilaterally, in the left middle frontal cortex, in the left superior temporal gyrus, in the left superior and right inferior occipital gyri, in the lingual gyri, bilaterally.

### Interaction effects

#### Task-by-age interactions

Three clusters, located in the left middle frontal and middle temporal cortex and in the left lingual gyrus, showed a task-by-age interaction effect (see Table 3). The former cluster was associated with reading-like tasks in children, the latter two clusters with reading-like tasks in adults.

**Table 3:** **Group-by-task, group-by-age, and task-by-age distribution of the activation peaks included in each of the four clusters showing significant interaction between two factors**.

		**Controls**	**Dyslexics**	
**LEFT MIDDLE TEMPORAL GYRUS (B.A. 37; CLUSTER ID: L86); *x* = −58, *y* = −58, *z* = 6**				
Task	Reading-like	14	0	14
	Non-reading-like	0	2	2
		14	2	16
			*p*-value	0.008
**LEFT SUPERIOR TEMPORAL GYRUS (B.A. 42; CLUSTER ID: L87); *x* = −60, *y* = −44, *z* = 16**				
Task	Reading-like	18	6	24
	Non-reading-like	0	3	3
		18	9	27
			*p*-value	0.028
**RIGHT INFERIOR PARIETAL LOBULE (B.A. 2; CLUSTER ID: R71); *x* = 50, *y* = −35, *z* = 48**				
Task	Reading-like	3	2	5
	Non-reading-like	0	7	7
		3	9	12
			*p*-value	0.045
**LEFT INFERIOR FRONTAL GYRUS, PARS OPERCULARIS (B.A. 44; CLUSTER ID: L57); *x* = −56, *y* = 15, *z* = 12**				
Age	Children	3	8	11
	Adults	12	2	14
		15	10	25
			*p*-value	0.004
**RIGHT INFERIOR FRONTAL GYRUS, PARS TRIANGULARIS (B.A. 45; CLUSTER ID: R57); *x* = 45, *y* = 32, *z* = 14**				
Age	Children	3	3	6
	Adults	10	0	10
		13	3	16
			*p*-value	0.036
**RIGHT PRECENTRAL GYRUS (B.A. 6; CLUSTER ID: R73); *x* = 47, *y* = −1, *z* = 34**				
Age	Children	7	0	7
	Adults	2	4	6
			4	13
			*p*-value	0.021
		**Children**	**Adults**	
**LEFT LINGUAL GYRUS (B.A. 18; CLUSTER ID: L84); *x* = −20, *y* = −88, *z* = −12**				
Task	Reading-like	11	15	26
	Non-reading-like	5	0	5
		16	15	31
			*p*-value	0.04
**LEFT MIDDLE TEMPORAL GYRUS (B.A. 21; CLUSTER ID: L14); *x* = −65, *Y* = −26, *z* = 6**				
Task	Reading-like	1	9	10
	Non-reading-like	4	2	6
		5	11	16
			*p*-value	0.04
**LEFT MIDDLE FRONTAL GYRUS (B.A. 44; CLUSTER ID: L44); *x* = −47, *y* = 16, *z* = 38**				
Task	Reading-like	13	2	15
	Non-reading-like	2	4	6
		15	6	21
			*p*-value	0.03

*x, y, and z refer to stereotactic coordinates of the centroid of each cluster*.

#### Group-by-task interactions

**Figure 5:**
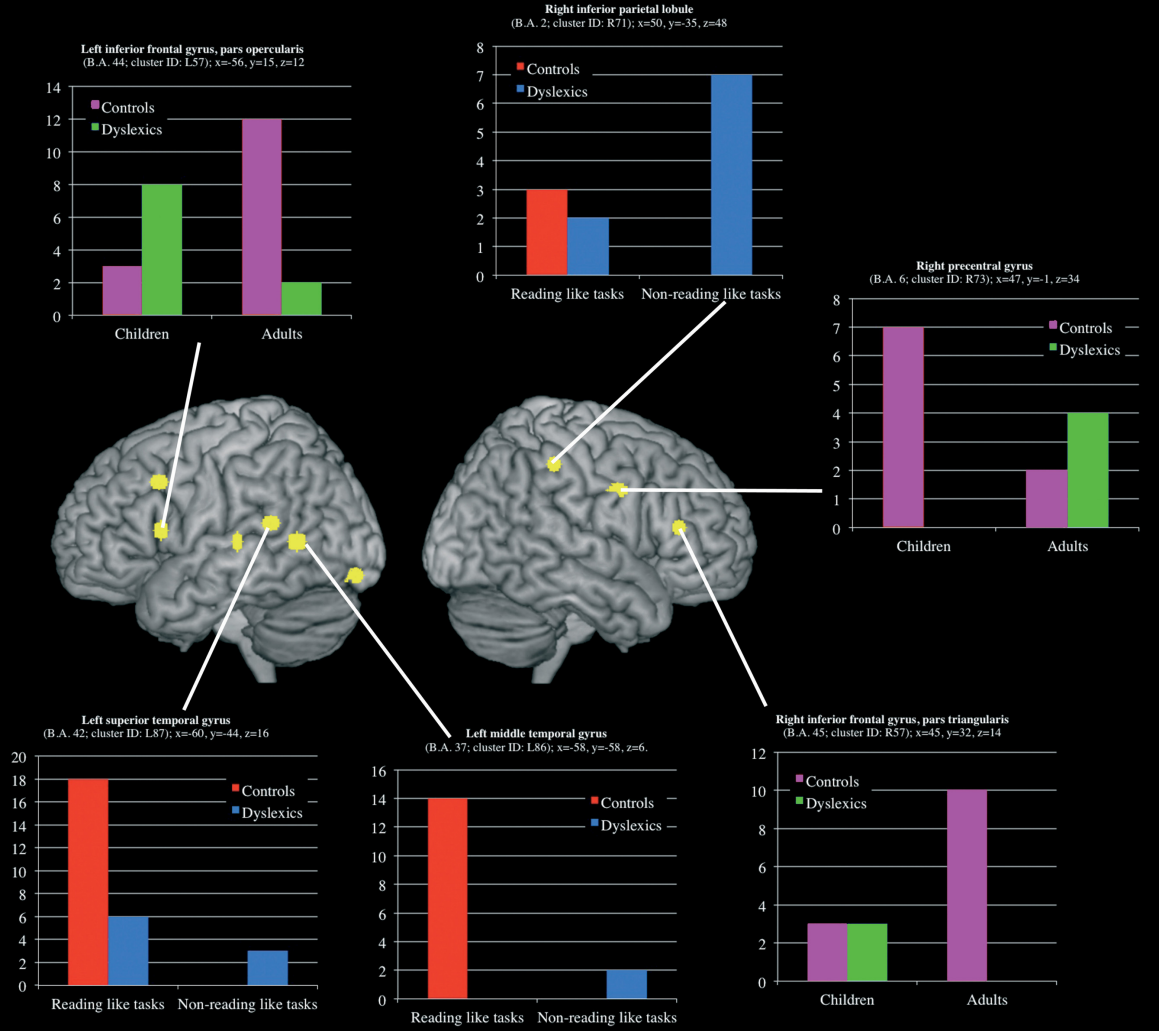
**Clusters that showed a significant group-by-task and group-by-age interaction effect**. For each clusters a histogram describe the peak distribution across group/conditions.

Three clusters, located in the left superior and middle temporal gyri and in the right inferior parietal lobule, showed a group-by-task interaction effect (see Table 3 and Figure 5 for details). The former two were associated with the normal controls for reading-like tasks, the third with the dyslexics for the non-reading-like tasks.

#### Group-by-age interactions

Three clusters, located in the opercular part of the left inferior frontal gyrus, in the triangular part of the right inferior frontal gyrus and in the right precentral gyrus, showed a group-by-age interaction effect (see Table 3 and Figure 5 for details). The former two clusters were associated with adult controls, the third with young controls.

### Undifferentiated clusters

Seven clusters, located in the pre-SMA, bilaterally, in the triangular part of the right inferior frontal gyrus, in the right insula, in the right middle cingulum, and in the right inferior occipital gyrus, did not showed a group-related preferential association (*p* > 0.5 in the binomial test) and were classified as undifferentiated activations (see Table 4 for details).

**Table 4:** **List of the undifferentiated clusters**.

**Cluster ID**	**Brain area**	***X***	***Y***	***Z***	***SD x***	***SD y***	***SD z***	**Controls**	**Dyslexics**
**UNDIFFERENTIATED CLUSTERS ACROSS NORMAL OR DYSLEXIC READERS GROUPS**									
R59	Right inferior occipital gyrus	23	−91	−2	5	6	5	10	7
R68	Right middle cingulum	10	26	33	6	5	5	11	8
R65	Right insula	34	21	−5	5	3	4	11	7
L90	Left inferior frontal gyrus, pars triangularis	−49	28	16	4	5	4	16	11
L47	Left SMA	−4	7	46	3	5	2	8	6
R52	Right SMA	2	3	65	3	4	3	7	4
R53	Right SMA	3	12	55	4	2	3	8	6

### Additional analyses

Because of the historical importance of the theories behind the scenes of the MT/V5 and cerebellar findings, *ad-hoc* special analysis was made for these two sets of findings.

#### MT/V5

**Figure 6:**
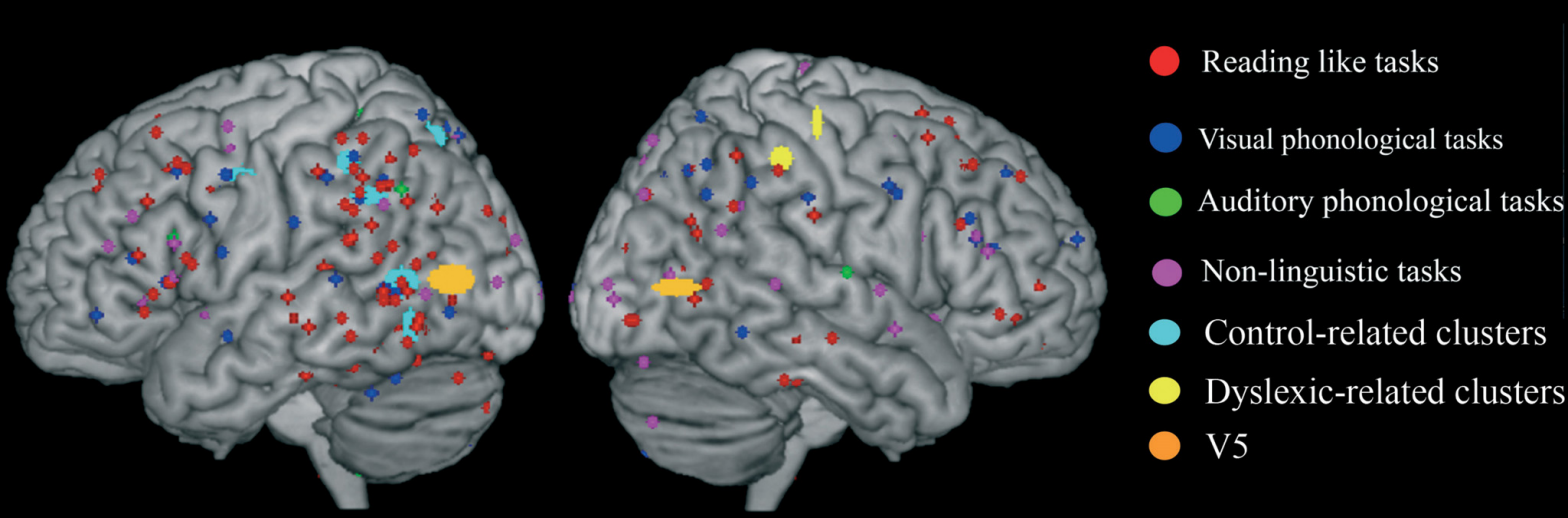
**Overlap of raw data, group-related clusters and the MT/V5 region of interest extracted from activations reported by Eden et al. (1996) in normal controls**.

We first identified “group” average stereotactic coordinates from Eden et al. (1996) from their eight normal subjects. This was done by using the same hierarchical clustering software of the meta-analysis. The centroid stereotactic locations of the MT/V5 region were located at *X* = -52; *Y* = -75; *Z* = 7; the SDs were: 11, 8, 5 mm in the three directions; on the right, the stereotactic coordinates were *X* = 50; *Y* = −70; *Z* = 5; the SDs were: 8, 8, 3 mm (areas in orange in Figure 6). As expected, Eden's et al. (1996) clusters fell within the statistical maps for visual motion perception described in Danelli et al. (2013).

We explored the anatomical congruence of these clusters with those that proved significant in the comparisons of controls and dyslexics in the meta-analysis. We also compared the Eden's MT/V5 location with the distribution of the raw data of the activations that were significantly larger in controls than in dyslexia. None of these analyses showed a systematic overlap of Eden's et al. (1996) MT/V5 and the data from other experiments on dyslexia.

We also compared the clusters associated with controls or dyslexics in the present study (see Table 2) with the mapping of the magnocellular system as identified by Danelli et al. (2013). There was one area of overlap (shared with an overlap for the motor learning task of Danelli et al., 2013) in one cluster located in the left superior parietal lobule (cluster L34). The experiments that generated these clusters in the data-set considered in this paper were based on phonological tasks, on a motor task in one case and on a visuo-spatial attentional tasks.

#### Cerebellum

**Figure 7:**
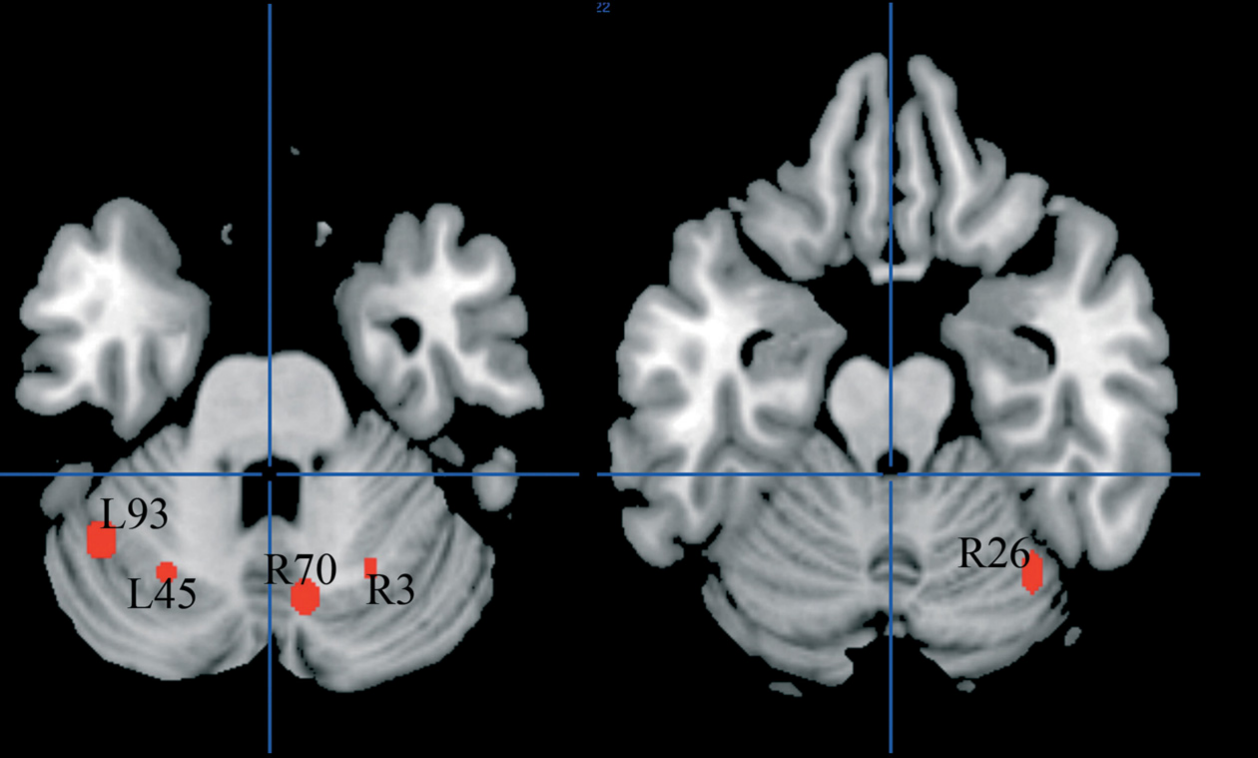
**Cerebellar clusters identified in both HC and ALE meta-analyses. In none of these there was a significant association with normal controls**.

There were five clusters identified by the general meta-analysis in the cerebellum (see Table 5 and Figure 7). These regions were identified by a variety of reading-like tasks (66 peaks overall) and non-reading-like tasks (11 peaks), with no specific association with either normal controls or developmental dyslexics for any of these clusters. Of the 77 peaks, only three peaks came from a comparison controls > dyslexics, 8 came from the comparison dyslexics > controls, 37 came from simple effects in the controls and 29 from simple effects in the dyslexics. Three of such clusters were significantly associated with data coming from adult volunteers.

**Table 5:** **Clusters emerged in the cerebellum**.

**Cluster I.D**.	**Area**	***X***	***Y***	***Z***	***SD x***	***SD y***	***SD z***	**Co/D**	***p***	**R/NR**	***p***	**Ch/A**	***P***
L45	Left cerebellum (lobule 6)	−26	−63	−27	4	3	3	5/9	n.s.	12/2	n.s.	2/12	0.007^A^
L93	Left cerebellum (crus I)	−42	−55	−30	4	5	4	15/9	n.s.	22/2	n.s.	5/19	0.004^A^
R70	Vermis (lobule 7)	8	−69	−27	4	5	4	12/9	n.s.	9/20	n.s.	1/20	<0.001^A^
R26	Right cerebellum (lobule 6)	34	−63	−19	3	6	4	6/5	n.s.	5/8	n.s.	2/9	0.063
R3	Right cerebellum (lobule 6)	24	−62	−31	2	3	4	2/5	n.s.	5/4	n.s.	2/5	n.s.

*Co, controls; D, Dyslexics; NR, non-reading-like tasks; R, reading-like tasks; A, Adults; Ch, Children*.

#### Meta-analysis restricted to the non-reading-like tasks

The hierarchical algorithm identified a total of 85 clusters with 2–10 peaks each, and had mean standard deviation along the three axes of 4.56 mm (x-axis), 5.21 mm (y-axis) and 5.09 mm (z-axis). After the comparison of these results with ALE maps, 40 out of 85 clusters were considered for subsequent analyses.

The *post-hoc* analyses identified only one cluster that could be associated with the normal controls to a statistically greater (*p* = 0.05) degree than with dyslexics: the cluster (centroid coordinates: *X* = 35; *y* = −41; *Z* = 41; the SDs were: 5, 6, 7 mm) was located in the right inferior parietal cortex. The cluster included peaks from motor tasks (#3), auditory perception tasks (#3) and three dimensional visual discrimination tasks (#3). The centroid of this cluster is very close to that of cluster R86 (centroid coordinates: *X* = 37; *y* = −45; *Z* = 43; the SDs were: 5, 5, 7 mm) of the general analysis in which again specificity for controls (i.e., lack of activation for dyslexics) was seen. This cluster was not observed in the ALE analysis.

## Discussion

The problem of co-occurrence of neural dysfunctions in dyslexia remained not explored by meta-analytic studies to date; it remained to be seen whether, besides the well replicated finding of a left occipito-temporal hypoactivation, there is a systematic co-occurrence of dysfunctional patterns of different functional systems, perhaps converging on the same brain regions associated with the reading deficit. Such evidence would be relevant for theories like, for example, the magnocellular or the cerebellar ones, which postulate a more basic and possibly more broadly distributed disorder in dyslexia.

In the present study this issue was tested by submitting to the meta-analysis all the suitable data^7^ from the literature on dyslexia, published up to September 2013, independently from the nature of the task, the materials, and the age-groups. Functional interpretation of the regional effects was made by direct exploration of the cluster compositions, by looking at the group or task that generated one effect, and appropriate *post-hoc* statistical tests, when possible, for broad categories. In addition, our evaluations of the results were also based on a direct comparison with the functional mapping of the reading, auditory phonological, visual magnocellular and visual motion, motor/cerebellar systems, and their intersections, as described by Danelli et al. (2013) for normal subjects using fMRI.

Our meta-analysis confirms one major milestone of previous empirical imaging studies and previous meta-analyses on dyslexia: the commitment to reading in normal controls for left occipito-temporal cortex (Paulesu et al., 2000; Cohen et al., 2002; Price et al., 2003), and the lack of such commitment in the same region for dyslexics (Shaywitz et al., 1998; Paulesu et al., 2001; Maisog et al., 2008; Richlan et al., 2009).

Because of the finer grained analysis afforded by our method, and thanks to the comparison with the independent fMRI data of Danelli et al. (2013), the same left occipito-temporal region identified by previous meta-analyses using ALE (Maisog et al., 2008; Richlan et al., 2009), was fractionated into three different clusters preferentially associated with the normal controls (L5, L6, and L23): clusters L5 and L23 are most likely associated with initial visual processing of the orthographic strings, while cluster L6 with the integration orthography with phonology.

By comparison with the data of Cohen et al. (2004) these corresponded to the visual-word form area (L5, VWFA), to the lateral inferior temporal multimodal region (L6; LIMA), and to an intermediate area (L23) that, to the best of our knowledge, has not been further characterized in the literature as yet.

There was more in our new data. The two more dorsal regions in the middle temporal and supra-marginal gyri (L86 and L89), were associated with the normal controls for a mixture of tasks including reading but also active phonological manipulation tasks, involving some working memory demands. These were not identified in the intersection paper of Danelli et al. (2013) most likely because the auditory phonological task had minimal demands in terms of manipulation and working memory processes.

Further up more dorsally, there was a new set of left hemispheric regions with different functional associations in parietal and premotor cortices and the supplementary motor area: these were brought about by a mix of reading, motor, phonological manipulation and visual attention tasks. Interestingly, once compared with Danelli et al. (2013) maps, some clusters overlapped with motoric regions (inferior parietal and SMA), while the left superior parietal lobule cluster overlapped with an intersection of motor learning and visual motion perception maps.

It is also worth noting that the meta-analysis restricted to the non-reading-like tasks revealed a right hemispheric inferior parietal cluster (R86; centroid coordinates: *X* = 37; *y* = −45; *Z* = 43; the SDs were: 5, 5, 7 mm) preferentially associated with normal readers, that is not present in developmental dyslexics. However, a more lateral right parietal cluster was preferential for the dyslexics (see Figure 4). There is overwhelming evidence of a role of the right parietal cortex in spatial attention (for review see Vallar et al., 2003; Corbetta and Shulman, 2011). The disorganized response in the right inferior parietal cortex of dyslexics (in some cases “more active,” in other cases “less active”) may be evidence for an anatomically grounded dysfunctional right hemispheric spatial attentional system in dyslexia.

On the other hand, no evidence was found either for a cerebellar dysfunction, nor for a left inferior frontal cortex hyperactivation in dyslexics, as in the previous meta-analyses (Maisog et al., 2008; Richlan et al., 2009).

In addition, we could not find evidence for the visual/magnocellular hypothesis of dyslexia, if this was to be benchmarked by a reduced recruitment of area V5/MT (Eden et al., 1996).

These findings expand previous evidence on the presence of functional anatomical deficits in dyslexia and identify a ventral to dorsal functional gradient with the more ventral areas, normally involved in the decoding aspect of reading (from orthography to phonology), the intermediate middle temporal and supra-marginal areas being related to reading-like behaviors or phonological processing and the more dorsal group being involved in reading but also in motoric or visual motion perception aspects of functional anatomy. We argue that the more dorsal left parietal and premotor cortex might be normally associated with eye-movement control or with visuo-spatial attention in language specific tasks. These would be functionally associated with the left hemispheric network of reading in normal controls but not in subjects with dyslexia.

This evidence brings new fuel for those believing in the existence of multiple dysfunctional systems in dyslexia without implying the need for focal and highly localized hypo-activations, preferentially associated with single classes of non-reading-like tasks. Rather, this new evidence speaks in favor of a distributed set of local malfunctions in “associative” regions normally involved in more than one behavior/cognitive domain. At a quantitative level, the number of peaks that contributed to the identification of these group-specific clusters was fairly balanced: 50 peaks for the occipito-temporal clusters, 31 for the intermediate network and 66 for the more dorsal network. It is worth noting that the more dorsal clusters appear group “specific”, or preferentially associated with normal readers, only if one considers the entire data-set rather than the non-reading-like behaviors on their own (data not shown). This result speaks in favor of our strategy of merging the data from multiple classes of tasks to reach a critical mass of observations.

### Comparison with the previous literature with particular reference to meta-analyses

There are other quantitative meta-analyses on the neural bases of developmental dyslexia in literature (Maisog et al., 2008; Richlan et al., 2009, 2011). In particular, these studies were focused on the task of reading or of reading-like behaviors, excluding auditory-verbal or non-linguistic tasks. Moreover, they included only peaks derived from group-by-task comparisons and used the ALE method (Maisog et al., 2008; Richlan et al., 2009). The potential advantages of our approach have been already commented upon.

Our findings are only partially consistent with the meta-analytic work published in literature. Indeed, control-related clusters emerged not only at the level of the left occipito-temporal and temporo-parietal regions, but also in the left middle temporal and parietal areas and in the supplementary motor cortex. Consistent with previous meta-analyses are also the more frequent subcortical effects for the dyslexics in the basal ganglia, for reading tasks.

Contrary to what described by Maisog et al. (2008) and Richlan et al. (2009), we could not find a reduced recruitment for the dyslexic group at the level of the left inferior frontal gyrus. However, it is worthy to note that a significant group-by-age interaction emerged in this area showing an association of this region with adult-control activations (as reported also by Richlan et al., 2011). The same area shows a “difficulty effect in phonological retrieval” in Cattinelli et al. (2013a) and Taylor et al. (2013) meta-analyses of reading, whereby the inferior frontal region is more active when reading non-words or low-frequency irregular words. An interaction effect emerged also for the right inferior frontal gyrus in the present study, while an association with control-children in the right precentral gyrus was observed.

A final difference with Richlan et al. (2011) is the lack of group by age interactions in the left parietal and occipito-temporal cortex. While not significant, technically speaking, at a statistical level, we note that an age effect in the left ventral occipito-temporal cortex was present and it was driven by adult normal readers (20 peaks for the controls, 1 peak for dyslexics) rather than by young readers (young controls: 6 peaks; young dyslexics: 0 peaks) and it is worth recalling that overall the number of “adult” and “young” peaks is balanced across the entire data-set. This is consistent with the idea that time is needed before the occipito-temporal cortex develops a neural expertise for reading (Dehaene et al., 2010).

Recently, Richlan et al. (2013) described a ALE-based meta-analysis of VBM data from dyslexia studies. The paper was mainly concerned with gray matter effects, as the papers that reported white matter abnormalities were only two (Eckert et al., 2005; Silani et al., 2005). The main trust of the paper is that there are reproducible reductions of gray matter in the left superior temporal sulcus; one of the coordinates described by them is consistent with the centroid of our cluster L86. The fact that functional imaging data show broader differences between normal controls and dyslexics when compared with the VBM ones, is a further argument in favor of the hypothesis that an abnormally wired cortex, rather than a focally damaged one, may better explain the functional disorder of dyslexia (see Silani et al., 2005, for further discussion; see also Paulesu et al., 1995; Klingberg et al., 2000).

### Visual magnocellular and cerebellar theories: chasing the wrong usual suspects?

As discussed in the introduction there is a non-negligible evidence of a visuo-perceptual deficit in children with dyslexia and some evidence for motoric deficits. The neural counterpart of these deficits has been sought by using visual motion perception tasks or motor learning tasks. The visual motion perception experiment of Eden et al. (1996) is the one that sits less comfortably with our results as we could not find a cluster in V5/MT, and of course, nor a group specific effect there. This difficulty may in part arise by the fact that the testing of the visual-magnocellular/attentional hypothesis has somewhat limited attention in the literature or by the fact that the main replication of the V5/MT finding was made using region of interest analyses (Demb et al., 1998), which were not included in our study.

Our attempt to test the V5 hypothesis by all means (see the results section) failed to identify a congruence with any of the effects described in the dyslexia literature, Eden et al. (1996) and Demb et al. (1998) excluded. However, our finding is consistent with more recent evidence on area V5. In a recent study, again based on a region of interest analysis of the data (preceded by a localizer experiment) Olulade et al. (2013) were able to show that if the dyslexics and the controls are equated for reading age rather than by age per-se, a significant difference in V5/MT cannot be found. A rehabilitation program on reading had a carry-over effect on V5/MT response (Olulade et al., 2013).

However, the visual magnocellular V5/MT hypothesis could be reformulated as a spatial attentional hypothesis. If so, activity in V5/MT may not be the best benchmark as discussed elsewhere (see Danelli et al., 2013, p. 2682). If the magnocellular hypothesis may still give an account for oculomotor control difficulties, there are better anatomical targets to be explored, for example, the dorsal premotor and parietal areas that we found less frequently activated in dyslexia. In the same vein, the evidence for a disorganized recruitment of the right inferior parietal lobule in dyslexia in non-linguistic tasks is a potentially revealing finding for all the theorists of attentional hypotheses in dyslexia.

Similar considerations apply for the cerebellar hypothesis. This could be easily reformulated in terms of deficient fine-grained motor control/learning without an a-priori commitment to the cerebellum. Indeed, none of the tasks whose deficit is attributed to the cerebellum by the believers in the cerebellar hypothesis, can be univocally and exclusively attributed to that organ: posture tasks, walking tasks, subtle finger coordination or bimanual tasks, motor learning tasks all depend on widely distributed neural systems in which the cerebellum is just one of the players (Kandel et al., 2012).

We found reduced recruitment of a series of motor regions in which there was a mixture of peaks derived from reading-like and non-reading-like tasks. Observation of these focal effects may contribute to a re-evaluation of motoric disorders in developmental dyslexia. Of course, fresh new experiments are needed to further address this hypothesis.

### The contribution of functional connectivity approaches and the disconnection hypothesis of dyslexia

Finally, our data could be discussed in the context of a more network based approach, such as that provided by functional or effective connectivity analyses. It has been repeatedly suggested that dyslexia could be associated with a failure of the functional interaction between distant brain regions that subserve diverse, perhaps elementary, cognitive operations needed for the task of reading and the like (Paulesu et al., 1995; Horwitz et al., 1998). These regions should have greater functional and effective connectivity in normal controls. Even though this disconnection hypothesis of dyslexia is particularly dear to us (Paulesu et al., 1995; Klingberg et al., 2000; Silani et al., 2005), the number of the connectivity studies is still limited and therefore our analysis was concentrated on classical studies based on univariate assessments of regional effects. There are two classes of such connectivity studies: task-based and resting state studies. Task-based studies reported reduced functional connectivity between reading-related areas like the left angular gyrus and the occipito-temporal cortex (Horwitz et al., 1998) or the occipito-temporal cortex and the frontal cortex (van der Mark et al., 2011; Finn et al., 2013; Schurz et al., 2014)^8^. In one study (Finn et al., 2013), a stronger right hemispheric connectivity for dyslexics was described. In the lone dynamic causal modeling (DCM) study performed on developmental dyslexia to date (Cao et al., 2008), reduced modulatory effects and connectivity were demonstrated in a temporo-parietal network for visual rhyming trials with conflicting orthography/phonology. It is worthy to note that task-based connectivity studies have an important limitation: the connectivity patterns explored are task dependent, the number of connections explored are limited in some cases (e.g., when using DCM), and different patterns could be produced by different reading tasks (see for example Levy et al., 2009); as a consequence, different dysfunctional patterns could emerge from the comparison between controls and dyslexics depending on the task under examination (Pugh et al., 2000). Resting-state connectivity studies, independent component analysis (ICA) studies (Wolf et al., 2010) or the technique proposed by Finn et al. (2013), may be more task-independent^9^ and better suited to test broader dysfunctions: while the ICA studies (Wolf et al., 2010) are difficult to interpret because one has to make assumptions on the functional meaning of the identified components and their comparability across different groups, the seed-based resting state functional connectivity studies have shown a reduction of connectivity between reading specific areas and regions not strictly involved in reading tasks, like, for example, between the left inferior parietal lobule and the left dorsal middle frontal areas (Koyama et al., 2013).

Taken together, these results are in line with the present findings, as they support the hypothesis that dyslexia could be the consequence of the co-occurrence of distributed dysfunctional patterns of different functional systems (see also Schurz et al., 2014): our data, however, also suggest a more limited degree of convergence of the multiple systems on high-level regions involved in reading-like as much as in non-reading-like tasks, particularly for the dorsal network identified here. Similar conclusions have not been made on the basis of a single study, even if based on a connectivity analysis. However, a more explicit demonstration of this general principle in the same sample of subjects is still in need.

## Conclusions

Taken together our results provide a partial reconciliation of different accounts of dyslexia, those more concerned with the decoding problem of dyslexia, the underlying phonological deficit and the deficit in the conversion from orthography to phonology, and those more focused on motoric and visuo-attentional problems. Interestingly, the more dorsally one moves within the system identified here, the more the contribution of non-reading-like tasks becomes relevant with a mixture of phonological awareness tasks and motoric/attentional tasks.

In at least one cluster, it was possible to make an indirect reference to a likely component of the magnocellular cortical network thanks to its intersection with the visuo-motor perception maps and motor learning maps of Danelli et al. (2013). The same cluster was observed in an independent meta-analysis on reading by Cattinelli et al. (2013a), the cluster being associated with reading tasks that are more demanding (e.g., as in pseudoword reading) because the stimuli seek greater visuo-attentional resources and require a finer grained control of eye-movement. The right inferior parietal cluster is also giving support to a multidimensional account of dyslexia. It would have been hard to make these conclusions on the basis of a single experiment or with a conventional meta-analysis based on ultra-specific and similar tasks. Yet, as we value the original contributions of the colleagues who produced the 53 papers submitted to a meta-analysis here, we urge the readers to refer to that original work for further discussions of the functional anatomical patterns of dyslexia.

### Conflict of interest statement

The authors declare that the research was conducted in the absence of any commercial or financial relationships that could be construed as a potential conflict of interest.

## Footnotes

^1^This labeling is used for convenience only without ideological positions about the importance of these deficits in dyslexia.

^2^One obvious historical such example can be the parietal region involved in phonological short-term memory: identified at the temporo-parietal junction by Paulesu et al. (1993, 1995), it was re-discovered, so to speak, two cm above by Smith et al. (1998). This is just one of the many obvious limitations of reviews based on qualitative approaches (for more discussions, see Fox et al., 1998; Cattinelli et al., 2013b).

^3^The rationale for using the two broad categories reading- and non-reading-like tasks, was dictated by the need to statistically assess the meaning of the clusters along a parsimonious, admittedly reduced, number of categories. The finer grained description of the more numerous yet broad classes of studies was left to the descriptive assessment of the composition of the clusters that proved significant for the two main categories. For example, once a cluster proved to be significantly associated with reading-like tasks, a qualitative exploration of the individual tasks that contributed to that effect allowed us to make some additional finer grained statement. See for example the case of clusters L5 and L6 in the results section.

^4^To make some justice of the fine-grained variability of the tasks that contributed to each cluster, beyond the two broad categories described above, each cluster was explored qualitatively (the raw data are available in the supplementary materials).

^5^In Danelli et al. (2013) reading was tested with a pseudoword silent reading task; the visual magnocellular/visual motion system with a visual motion perception task using Gabor low-frequency patches; the auditory phonological system was tested with a rhyming task for syllabic sounds; the motor cerebellar system was tested with a finger tapping sequence learning task.

^6^The idea here is that the cloud of peaks from the reading-like experiments may operate as geometrical attractors in the clustering procedure and mask spatial effects coming from the less numerous data-set from non-reading-like experiments. The separate meta-analysis should have protected us from this hypothetical confound.

^7^See inclusion criteria for the raw data in the methods section.

^8^Finn et al. (2013) is a task-based study in which the weight of the task was regressed out. There are studies, however, in which reduced of connectivity was not found in dyslexics (Richards and Berninger, 2008). Here, an enhanced connectivity between the left inferior frontal gyrus and the right homolog was reported.

^9^The resting state condition, with the vaigue instructions attached, remains a task on its own.

## Supplementary material

Table S1 **For each peak we reported the MNI coordinates (MNIx,y,z), the name of the first author, the journal and the year of publication of the article, the technique (PET or fMRI) and the stereotactic space used, the mean age of participants and the composition of the sample, the nature of the task, the nature of the functional contrast from which the peak was extracted**. Moreover, we reported, for each peak, the corresponding cluster ID and the cluster label. In particular, for each cluster we reported the coordinates of the centroid (Mean MNIx,y,z) and the standard deviation on the three axes (SDx,y,z), the corresponding anatomical area. Finally, we reported, for each peak, the binomial classifications (non-reading or reading-like task; children or adults, controls or dyslexics) used for the *post-hoc* analyses.

Table S2 **Peak distribution in group-related clusters**.
